# The Accuracy of a Method for Printing Three-Dimensional Spinal Models

**DOI:** 10.1371/journal.pone.0124291

**Published:** 2015-04-27

**Authors:** Ai-Min Wu, Zhen-Xuan Shao, Jian-Shun Wang, Xin-Dong Yang, Wan-Qing Weng, Xiang-Yang Wang, Hua-Zi Xu, Yong-Long Chi, Zhong-Ke Lin

**Affiliations:** 1 Department of Orthopaedics, Second Affiliated Hospital of Wenzhou Medical University, Zhejiang Spinal Research Center, 109# Xue Yuan Western Road, Wenzhou, Zhejiang, People’s Republic of China; 2 Department of Anatomy, Wenzhou Medical University, Higher Education Zone, Wenzhou, Zhejiang, People’s Republic of China; Shenzhen institutes of advanced technology, CHINA

## Abstract

**Background:**

To study the morphology of the human spine and new spinal fixation methods, scientists require cadaveric specimens, which are dependent on donation. However, in most countries, the number of people willing to donate their body is low. A 3D printed model could be an alternative method for morphology research, but the accuracy of the morphology of a 3D printed model has not been determined.

**Methods:**

Forty-five computed tomography (CT) scans of cervical, thoracic and lumbar spines were obtained, and 44 parameters of the cervical spine, 120 parameters of the thoracic spine, and 50 parameters of the lumbar spine were measured. The CT scan data in DICOM format were imported into Mimics software v10.01 for 3D reconstruction, and the data were saved in .STL format and imported to Cura software. After a 3D digital model was formed, it was saved in Gcode format and exported to a 3D printer for printing. After the 3D printed models were obtained, the above-referenced parameters were measured again.

**Results:**

Paired t-tests were used to determine the significance, set to P<0.05, of all parameter data from the radiographic images and 3D printed models. Furthermore, 88.6% of all parameters of the cervical spine, 90% of all parameters of the thoracic spine, and 94% of all parameters of the lumbar spine had Intraclass Correlation Coefficient (ICC) values >0.800. The other ICC values were <0.800 and >0.600; none were <0.600.

**Conclusion:**

In this study, we provide a protocol for printing accurate 3D spinal models for surgeons and researchers. The resulting 3D printed model is inexpensive and easily obtained for spinal fixation research.

## Introduction

The study of anatomy is an important part of medical education. Furthermore, with a sound knowledge of anatomy, we can design new surgical fixation techniques. A good example of spinal surgery is the pedicle screw fixation technique, which was rarely used before 1907 [1]. However, as more anatomic features of this technique were described, spine surgeons began to attempt the technique, and it has since become widely used in thoracic, lumbar, and sacral regions [2, 3].

To study the morphology of the human spine and new spinal fixation methods, scientists require cadaveric specimens, which are dependent on donation. However, in most countries, the number of people willing to donate their body is low [4]. Indeed, some religions and faiths discourage people from donating their body, and as a result, access to cadaveric specimens is very limited in some countries.

Without sufficient numbers of cadaveric specimens, some researchers learn new techniques only by studying radiographic images [5–8]. Computed tomographic analysis may be sufficient for morphometric study, but the use of cadaveric specimens is preferable and more reliable [9–11]. Moreover, cadaveric research is necessary for some studies.

3D printing is a process for making a 3D-printed model of almost any shape from a 3D digital model or other electronic data source [12]. The 3D printing methods include selective laser melting, laser sintering, fused deposition modeling, stereolithography, laminated object manufacturing and fused filament fabrication [13, 14]. Many materials are used in these different 3D printing techniques; e.g. thermoplastics (PLA and ABS) are commonly used for the fuse filament fabrication technique, where as titanium alloys and cobalt chrome alloys are used for the selective laser melting technique. Researchers can choose different materials according to the 3D printing technique to be used and the properties, cost and color of materials that they prefer [12, 15, 16].

We previously reconstructed a 3D digital spinal model from CT scan data, and it has been shown that this 3D digital model is morphologically accurate [17]. Accordingly, an accurate 3D model printed from a 3D-reconstructed digital model would allow for studying the morphological features of the 3D printed model, which may solve the problem of scarce cadaveric specimens.

## Materials and Methods

This study was performed following the Declaration of Helsinki principles and was approved by the Institutional Review Board (IRB) of The Second Affiliated Hospital of Wenzhou Medical University. Written informed consent was obtained from all participants.

Forty-five computed tomography (CT) scans of cervical, thoracic or lumbar spines of patients (mean age 42.5±7.7 years (range 31–54 years))were obtained using the Star PACS system (INFINITT, Seoul, South Korea) of our hospital. The included cervical, thoracic and lumbar spines lacked spinal disease, as shown by CT scans for health examination or because the patients had presented with oral, anterior neck, cardiac, pulmonary or abdominal diseases. Patients with any spinal abnormality, such as fracture, scoliosis or tumor, were excluded.

**Fig 1:**
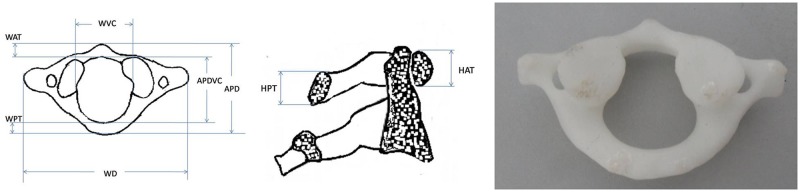
Schematic diagram showing the method of measurement for C1 (Atlas). **WD** is the abbreviation of Width diameter; **APD** is Anteroposterior diameter; **WVC** is Width of vertebral canal; **APDVC** is Anteroposterior diameter of vertebral canal; **WAT** is Width of anterior tubercle; **HAT** is Height of anterior tubercle; **WPT** is Width of posterior tubercle; and **HPT** is Height of posterior tubercle.

We measured the following parameters using the Star PACS System, which was proven to achieve accurate measurements in previous studies [8, 18]:

**C1** (Fig 1): Width diameter; Anteroposterior diameter; Width of vertebral canal; Anteroposterior diameter of vertebral canal; Width of anterior tubercle; Height of anterior tubercle; Width of posterior tubercle; Height of posterior tubercle.

**Fig 2:**
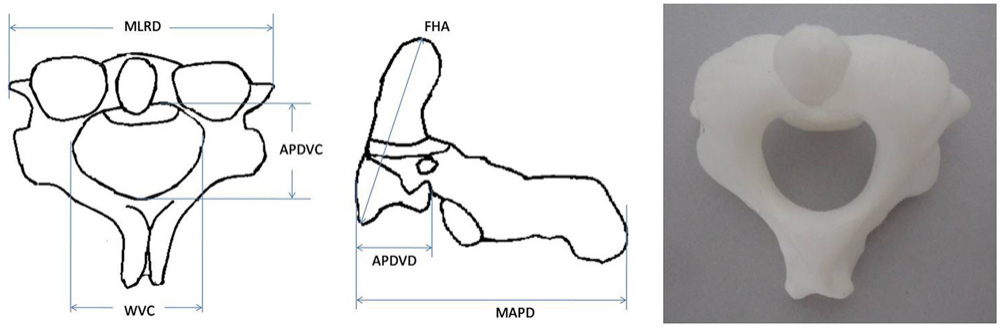
Schematic diagram showing the method of measurement for C2 (Axis). **MAPD** is the abbreviation of Max anteroposterior diameter; **MLRD** is Max left-right diameter; **APDVD** is Anteroposterior diameter of vertebral body; **WVC** is Width of vertebral canal; **APDVC** is Anteroposterior diameter of vertebral canal; and **FHA** is Frontal height of axis (including the odontoid process).

**C2** (Fig 2): Max Anteroposterior diameter; Max left-right diameter; Anteroposterior diameter of vertebral body; Width of vertebral canal; Anteroposterior diameter of vertebral canal; Frontal height of axis (including odontoid process).

**Fig 3:**
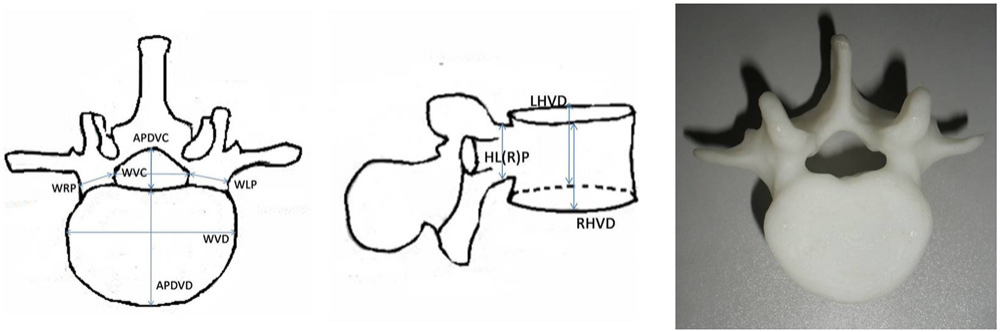
Schematic diagram showing the method for measurement ofC3-L5. **WVD** is the abbreviation of Width of vertebral body; **APDVD** is Anteroposterior diameter of vertebral body; **LHVD** is Left height of vertebral body; **RHVD** is Right height of vertebral body; **WVC** is Width of vertebral canal; **APDVC** is Anteroposterior diameter of vertebral canal; **WRP** is Width of right pedicle; **HRP** is Height of right pedicle; **WLP** is Width of left pedicle; **HLP** is Height of left pedicle.

**C3-L5** (Fig 3): Width of vertebral body; Anteroposterior diameter of vertebral body; Left height of vertebral body; Right height of vertebral body; Width of vertebral canal; Anteroposterior diameter of vertebral canal; Width of right pedicle; Height of right pedicle; Width of left pedicle; Height of left pedicle. Because the pedicles of the cervical spine are very small, Width of right pedicle, Height of right pedicle, Width of left pedicle, and Height of left pedicle were not measured at C3–C7.

The CT scan data were then imported in DICOM format into Mimics software v10.01 (Materialise, Leuven, Belgium)for 3D reconstruction. The threshold value was set at “Bone (CT)”, “226-Max”, which is optimal for bone reconstruction. After the 3D digital images were calculated and reconstructed, we removed the bone, which was not needed, and every vertebra was then separated. The data were then saved in STL format and imported into Cura software. After a 3D digital model was formed, we saved it in Gcode format and exported it to a3D printer (3D ORTHO Waston Med Inc. Changzhou, Jiangsu, China) to print the objects. The scale was set at 1:1, and PLA (Polylactic acid:(C_3_H_4_O_2_)_n_), with a molecular weight of 5000–700000 according to the product instruction, was used as the print material. After the 3D-printed models were obtained, the above-mentioned parameters were measured again.

### Statistical analysis

The data were analyzed using the SPSS software (version 17.0, SPSS Inc., Chicago, IL, USA). Comparisons of the radiographic image and 3D-printed model data were made using paired t-tests, with the level of significance set at P<0.05. If P>0.05, the Intraclass Correlation Coefficient (ICC) was calculated to assess how strongly the data from radiographic images and 3D printed models resembled each other.

## Results

Forty-four parameters of the cervical spine, 120 parameters of the thoracic spine, and 50 parameters of the lumbar spine were measured.

For C1, the respective values of WD, APD, WVC, APDVC, WAT, HAT, WPT, and HPT were 76.29±4.43, 43.23±2.37, 28.84±1.87, 27.90±1.73, 7.66±0.98, 12.22±0.81, 7.74±1.08, and 11.76±1.27 m min the radiographic images and 76.33±4.20, 43.15±2.37, 28.68±1.69, 28.01±1.75, 7.53±1.18, 12.13±1.12, 7.88±1.06, and11.63±1.29 m min the 3D-printed models (Table 1). For C2, the respective values of MAPD, MLRD, APDVD, WVC,APDVC, and FHA were 44.69±1.86, 52.46±1.99, 13.24±1.32, 22.66±1.12, 16.23±1.65, and 37.45±2.74 m min the radiographic images and 44.73±1.91, 52.54±2.02,13.36±1.40, 22.51±0.96, 16.39±1.58, and 37.63±2.68 m min the 3D-printed models (Table 2). For C3–C7, the WVD, APDVD, WVC, APDVC, RHVD, and LHVD parameters of C3 were21.86±1.33, 16.35±1.14, 22.72±1.30, 13.18±0.97,13.85±1.15, and 13.91±1.08 mm, respectively, in the radiographic images, and most of them gradually increased by C7. A similar trend was found in the 3D models (Table 3).

**Table 1:** The atlas parameters and comparison of data from radiographic images and 3D-printed models.

	WD	APD	WVC	APDVC	WAT	HAT	WPT	HPT
Radiographic image	76.29±4.43	43.23±2.37	28.84±1.87	27.90±1.73	7.66±0.98	12.22±0.81	7.74±1.08	11.76±1.27
Printed model	76.33±4.20	43.15±2.37	28.68±1.69	28.01±1.75	7.53±1.18	12.13±1.12	7.88±1.06	11.63±1.29
T	-0.299	1.084	1.748	-1.201	1.774	1.103	-1.644	1.793
P	0.766	0.284	0.087	0.236	0.083	0.276	0.107	0.080
ICC	0.977	0.976	0.944	0.933	0.882	0.847	0.851	0.927

**Note**: **WD:** Width diameter; **APD:** Anteroposterior diameter; **WVC:** Width of vertebral canal; **APDVC:** Anteroposterior diameter of vertebral canal; **WAT:** Width of anterior tubercle; **HAT:** Height of anterior tubercle; **WPT:** Width of posterior tubercle; **HPT**: Height of posterior tubercle.

**Table 2:** The axis parameters and comparison of data from radiographic images and 3D-printed models.

	MAPD	MLRD	APDVD	WVC	APDVC	FHA
Radiographic image	44.69±1.86	52.46±1.99	13.24±1.32	22.66±1.12	16.23±1.65	37.45±2.74
Printed model	44.73±1.91	52.54±2.02	13.36±1.40	22.51±0.96	16.39±1.58	37.63±2.68
T	-0.336	-0.673	-1.247	1.570	-1.589	-1.439
P	0.739	0.504	0.219	0.123	0.119	0.157
ICC	0.928	0.922	0.878	0.799	0.914	0.952

**Note: MAPD**: Max anteroposterior diameter; **MLRD**: Max left-right diameter; **APDVD**: Anteroposterior diameter of vertebral body; **WVC**: Width of vertebral canal; **APDVC**: Anteroposterior diameter of vertebral canal; **FHA**: Frontal height of axis (Including odontoid process).

**Table 3:** The parameters of C3–C7 and comparison of data from radiographic images and 3D-printed models.

	WVD	APDVD	WVC	APDVC	RHVD	LHVD
**C3**						
Radiographic image	21.86±1.33	16.35±1.14	22.72±1.30	13.18±0.97	13.85±1.15	13.91±1.08
Printed model	22.03±1.15	16.25±0.93	22.61±1.29	13.34±0.94	13.75±1.20	13.81±0.95
T	-1.549	0.901	0.980	-1.607	1.478	1.063
P	0.129	0.373	0.333	0.115	0.146	0.293
ICC	0.830	0.766	0.813	0.746	0.926	0.834
**C4**						
Radiographic image	22.44±1.38	16.35±1.08	23.10±1.26	12.78±0.89	13.31±1.17	13.56±1.05
Printed model	22.27±1.13	16.38±1.18	23.10±1.18	12.82±0.84	13.32±0.89	13.38±0.99
T	1.515	-0.496	0.019	-0.944	-0.102	1.553
P	0.137	0.622	0.985	0.351	0.920	0.128
ICC	0.830	0.948	0.949	0.935	0.816	0.706
**C5**						
Radiographic image	26.56±1.30	17.43±1.08	22.56±1.39	12.83±0.72	14.84±0.82	14.79±1.02
Printed model	26.70±1.09	17.34±1.35	22.45±1.28	12.78±0.62	14.72±0.90	14.66±1.07
T	-1.615	1.037	0.944	1.380	1.551	1.399
P	0.113	0.305	0.350	0.175	0.128	0.169
ICC	0.879	0.905	0.830	0.918	0.824	0.822
**C6**						
Radiographic image	28.49±1.50	18.28±1.26	24.41±1.22	13.40±0.77	16.41±0.84	16.17±1.10
Printed model	28.55±1.64	18.15±1.29	24.48±1.04	13.35±0.76	16.36±0.94	16.10±0.83
T	-0.531	1.309	-0.922	1.425	0.74	0.998
P	0.598	0.197	0.361	0.161	0.463	0.324
ICC	0.883	0.856	0.915	0.954	0.843	0.910
**C7**						
Radiographic image	29.70±1.18	18.95±1.13	22.84±1.43	14.34±0.71	16.78±0.96	16.66±1.15
Printed model	29.52±1.24	18.82±1.04	22.99±1.40	14.31±0.63	16.79±0.96	16.60±1.12
T	1.628	1.323	-1.480	0.712	-0.078	1.275
P	0.111	0.193	0.146	0.480	0.938	0.209
ICC	0.799	0.814	0.874	0.890	0.942	0.970

**Note: WVD**: Width of vertebral body; **APDVD**: Anteroposterior diameter of vertebral body; **WVC**: Width of vertebral canal; **APDVC**: Anteroposterior diameter of vertebral canal; **RHVD**: Right height of vertebral body; **LHVD**: Left height of vertebral body.

The respective WVD, APDVD, LHVD, RHVD, WVC, APDVC, WRP, HRP, WLP, HLPT parameters of T1 were 31.36±1.41, 18.88±1.24, 19.50±0.87, 19.40±1.13, 21.44±1.16, 14.50±0.61, 9.35±0.83, 10.51±0.68, 9.48±0.55, and 10.33±0.85 mm in the radiographic images, and most of them gradually increased by L5 (46.44±2.64, 32.43±2.03, 13.41±1.39, 13.12±1.31, 26.27±1.46, 26.36±1.44, 32.13±2.22, 17.73±1.96, 16.91±1.60, and 17.24±1.97 mm, respectively). We found that the 3D printed model data also showed similar trends (Table 4 **and** Table 5).

**Table 4:** The parameters of T1–T12 and comparison of data from radiographic images and 3D-printed models.

	WVD	APDVD	LHVD	RHVD	WVC	APDVC	WRP	HRP	WLP	HLP
**T1**										
Radiographic image	31.36±1.41	18.88±1.24	19.50±0.87	19.40±1.13	21.44±1.16	14.50±0.61	9.35±0.83	10.51±0.68	9.48±0.55	10.33±0.85
Printed model	31.25±1.30	18.93±1.20	19.55±0.84	19.46±1.04	21.31±1.11	14.42±0.69	9.31±0.86	10.56±0.74	9.54±0.58	10.43±0.74
T	1.314	-0.709	-1.289	-0.701	1.332	1.302	0.599	-0.902	-1.376	-1.425
P	0.196	0.482	0.204	0.487	0.190	0.200	0.552	0.372	0.176	0.161
ICC	0.913	0.927	0.953	0.877	0.841	0.755	0.869	0.835	0.882	0.819
**T2**										
Radiographic image	32.29±1.17	20.80±1.15	19.16±0.91	19.04±0.72	17.69±0.95	14.60±0.74	7.72±0.67	11.50±0.83	8.05±0.71	11.62±0.82
Printed model	32.23±1.26	20.71±1.19	19.09±1.00	19.13±0.84	17.55±1.05	14.63±0.63	7.78±0.66	11.63±0.80	7.93±0.68	11.77±0.91
T	0.833	0.960	0.861	-1.672	1.612	-0.577	-1.442	-1.692	1.684	-1.731
P	0.410	0.342	0.394	0.102	0.114	0.567	0.156	0.098	0.099	0.090
ICC	0.911	0.862	0.801	0.878	0.837	0.908	0.934	0.823	0.764	0.782
**T3**										
Radiographic image	28.74±1.20	21.11±1.36	17.36±0.96	17.69±0.91	16.09±1.17	14.51±0.75	5.68±0.63	14.07±1.00	5.85±0.66	14.00±0.92
Printed model	28.83±1.17	20.94±1.14	17.24±0.87	17.62±0.90	15.96±0.94	14.58±0.66	5.62±0.63	14.19±0.64	5.78±0.60	14.11±0.82
T	-1.402	1.538	1.383	0.933	1.264	-1.292	1.190	-1.664	1.309	-1.785
P	0.168	0.131	0.174	0.356	0.213	0.203	0.240	0.103	0.197	0.081
ICC	0.932	0.813	0.808	0.810	0.787	0.885	0.874	0.834	0.870	0.894
**T4**										
Radiographic image	29.15±1.16	24.11±1.17	18.06±1.00	18.27±0.77	16.03±0.92	14.71±0.77	5.16±0.65	13.92±0.85	5.47±0.71	13.62±1.03
Printed model	29.31±1.30	24.23±1.13	17.95±0.91	18.36±0.88	15.95±0.94	14.63±0.85	5.09±0.69	14.09±0.73	5.36±0.75	13.81±0.93
T	-1.627	-1.423	1.632	-1.703	1.576	1.035	1.083	-1.693	1.635	-1.845
P	0.111	0.162	0.110	0.096	0.122	0.306	0.285	0.097	0.109	0.072
ICC	0.872	0.89	0.894	0.910	0.937	0.813	0.817	0.655	0.810	0.735
**T5**										
Radiographic image	27.56±1.39	26.7±1.46	19.74±0.82	19.61±0.77	15.82±1.23	14.80±0.87	5.75±0.60	13.67±0.76	5.3±0.62	13.62±0.85
Printed model	27.47±1.33	26.79±1.29	19.68±0.75	19.61±0.79	15.74±1.30	14.85±0.67	5.64±0.63	13.76±0.79	5.34±0.55	13.71±0.81
T	1.173	-1.043	1.317	-0.062	1.247	-1.038	1.778	-1.737	-0.82	-1.742
P	0.247	0.303	0.195	0.951	0.219	0.305	0.082	0.089	0.417	0.088
ICC	0.923	0.909	0.937	0.932	0.945	0.890	0.789	0.899	0.840	0.907
**T6**										
Radiographic image	29.01±1.32	27.73±1.63	20.25±1.11	20.06±1.19	15.43±0.84	14.64±0.66	6.12±0.7	13.49±0.87	5.97±0.66	13.74±1.02
Printed model	29.19±1.26	27.51±1.30	20.18±1.04	19.99±1.02	15.46±0.91	14.71±0.68	6.02±0.73	13.63±0.87	5.89±0.47	13.84±1.03
T	-1.605	1.557	0.904	1.612	-0.788	-1.675	1.45	-1.814	1.639	-1.707
P	0.116	0.127	0.371	0.114	0.435	0.101	0.154	0.076	0.108	0.095
ICC	0.819	0.808	0.901	0.962	0.952	0.928	0.802	0.812	0.828	0.924
**T7**										
Radiographic image	30.10±1.35	28.78±1.6	20.48±1.15	19.92±1.06	15.22±0.99	14.77±0.86	5.97±0.77	13.57±0.89	5.89±0.64	13.9±1.02
Printed model	30.26±1.39	28.66±1.28	20.61±1.28	19.81±1.09	15.32±1.09	14.87±0.79	5.88±0.75	13.73±0.85	5.82±0.60	13.96±1.03
T	-1.623	0.988	-1.666	1.618	-1.020	-1.644	1.389	-1.627	1.275	-0.698
P	0.112	0.329	0.103	0.113	0.313	0.107	0.172	0.111	0.209	0.489
ICC	0.875	0.836	0.913	0.906	0.815	0.870	0.844	0.727	0.804	0.847
**T8**										
Radiographic image	31.33±1.34	27.66±1.16	20.85±1.11	20.91±1.05	15.95±1.06	14.52±0.72	6.29±0.78	13.25±0.69	6.36±0.66	13.26±0.78
Printed model	31.25±1.16	27.81±1.11	20.91±0.95	20.77±1.21	15.85±1.07	14.58±0.64	6.20±0.67	13.33±0.66	6.42±0.64	13.22±0.73
T	0.858	-1.872	-1.36	1.594	1.688	-1.059	1.541	-1.224	-1.642	0.544
P	0.396	0.068	0.181	0.118	0.098	0.295	0.130	0.227	0.108	0.387
ICC	0.883	0.89	0.947	0.855	0.923	0.850	0.850	0.764	0.929	0.918
**T9**										
Radiographic image	32.07±1.41	30.37±1.46	20.75±1.18	21.16±1.12	16.12±1.08	14.39±0.69	6.45±0.64	13.96±0.64	6.30±0.67	14.08±0.66
Printed model	32.23±1.04	30.54±1.59	20.87±1.26	21.07±1.14	15.98±1.08	14.48±0.79	6.35±0.72	14.05±0.64	6.27±0.73	14.15±0.66
T	-1.724	-1.673	-1.588	1.442	1.617	-1.425	1.573	-1.354	0.466	-1.716
P	0.092	0.101	0.12	0.156	0.113	0.161	0.123	0.183	0.643	0.093
ICC	0.880	0.898	0.916	0.928	0.866	0.828	0.819	0.738	0.820	0.920
**T10**										
Radiographic image	33.39±1.40	28.09±1.16	21.53±1.13	21.13±1.12	16.09±1.17	14.94±0.81	7.44±0.61	16.71±0.96	7.47±0.66	16.77±0.88
Printed model	33.29±1.22	27.98±1.12	21.38±1.16	20.95±1.23	16.21±1.12	15.02±0.92	7.39±0.64	16.80±0.90	7.45±0.64	16.83±0.93
T	0.896	1.508	1.667	1.543	-1.636	-1.208	1	-1.435	0.360	-0.986
P	0.375	0.139	0.103	0.130	0.109	0.207	0.174	0.158	0.721	0.330
ICC	0.839	0.919	0.849	0.778	0.896	0.885	0.930	0.884	0.868	0.896
**T11**										
Radiographic image	33.96±1.40	27.50±1.21	21.68±1.17	22.42±1.12	17.20±1.07	16.18±0.80	8.28±0.70	18.48±0.81	8.08±0.77	18.26±1.02
Printed model	33.85±1.24	27.36±1.19	21.84±1.26	22.32±1.13	17.33±1.08	16.29±0.79	8.26±0.75	18.56±0.75	7.97±0.71	18.31±0.97
T	1.214	1.806	-1.689	1.671	-1.705	-1.588	0.268	-1.375	1.815	-0.623
P	0.231	0.078	0.098	0.102	0.095	0.128	0.790	0.176	0.076	0.537
ICC	0.897	0.913	0.867	0.933	0.882	0.815	0.81	0.900	0.834	0.884
**T12**										
Radiographic image	37.66±1.75	27.39±1.33	23.43±1.30	23.36±1.32	17.79±1.17	16.95±0.91	8.02±0.71	17.01±0.91	7.89±0.75	17.02±0.97
Printed model	37.71±1.64	27.22±1.09	23.38±1.34	23.30±1.27	17.73±1.28	17.05±0.92	7.96±0.68	17.01±0.66	7.76±0.63	17.05±0.80
T	-0.569	1.662	0.610	0.893	0.566	-1.381	1.582	-0.071	1.748	-0.505
P	0.572	0.104	0.545	0.376	0.574	0.174	0.121	0.943	0.087	0.616
ICC	0.928	0.843	0.926	0.937	0.879	0.877	0.937	0.849	0.726	0.916

**Note**: **WVD**: Width of vertebral body; **APDVD**: Anteroposterior diameter of vertebral body; **LHVD**: Left height of vertebral body; **RHVD**: Right height of vertebral body; **WVC**: Width of vertebral canal; **APDVC**: Anteroposterior diameter of vertebral canal; **WRP**: Width of right pedicle; **HRP**: Height of right pedicle; **WLP**: Width of left pedicle; **HLP**: Height of left pedicle.

**Table 5:** The parameters of L1–L5 and comparison of data from radiographic images and 3D-printed models.

	WVD	APDVD	LHVD	RHVD	WVC	APDVC	WRP	HRP	WLP	HLP
**L1**										
Radiographic image	36.95±1.49	28.66±1.67	25.13±1.37	24.81±1.27	22.14±1.43	16.10±1.09	7.92±0.65	15.61±0.86	8.10±0.68	15.73±0.81
Printed model	37.08±1.45	28.47±1.62	24.99±1.42	24.75±1.21	22.27±1.31	16.23±1.05	8.01±0.74	15.51±0.90	8.04±0.69	15.65±0.89
T	-1.306	1.743	1.116	0.640	-1.117	-1.469	-1.616	1.724	0.923	1.130
P	0.198	0.088	0.271	0.525	0.270	0.149	0.113	0.092	0.361	0.264
ICC	0.885	0.901	0.813	0.861	0.822	0.844	0.847	0.891	0.775	0.859
**L2**										
Radiographic image	38.30±1.35	29.20±1.55	26.09±1.65	26.20±1.58	22.53±1.68	16.49±1.27	8.60±1.46	15.34±0.81	8.37±1.17	15.14±1.10
Printed model	38.21±1.49	29.28±1.63	25.96±1.68	26.07±1.88	22.68±1.69	16.61±1.40	8.39±1.59	15.26±0.9	8.43±1.31	15.23±1.05
T	1.220	-1.301	1.102	1.026	-1.513	-1.371	1.792	1.434	-0.585	-1.396
P	0.229	0.200	0.276	0.310	0.137	0.137	0.080	0.158	0.562	0.169
ICC	0.939	0.961	0.890	0.887	0.922	0.902	0.871	0.897	0.857	0.917
**L3**										
Radiographic image	39.89±2.05	30.48±1.78	25.37±1.68	25.30±1.65	22.47±2.16	15.68±1.65	9.97±1.44	14.86±1.51	9.91±1.40	14.93±1.40
Printed model	39.73±2.12	30.34±1.85	25.53±1.66	25.16±1.66	22.59±2.04	15.81±1.76	10.06±1.55	14.98±1.51	9.77±1.46	15.03±1.48
T	1.586	1.647	-1.798	1.144	-1.443	-1.33	-0.906	-1.191	1.154	-1.018
P	0.120	0.107	0.079	0.259	0.156	0.190	0.370	0.240	0.255	0.314
ICC	0.964	0.956	0.939	0.895	0.950	0.935	0.887	0.891	0.845	0.842
**L4**										
Radiographic image	41.93±1.40	31.40±1.15	27.52±1.36	27.33±1.74	24.22±1.93	15.91±1.45	11.34±1.51	14.28±1.26	10.75±1.47	14.18±0.96
Printed model	42.02±1.35	31.50±1.27	27.39±1.41	27.46±1.56	23.97±1.52	15.85±1.42	11.25±1.50	14.24±1.31	10.97±1.41	14.36±1.09
T	-1.029	-0.811	1.606	-1.508	1.683	0.611	1.149	0.501	-1.702	-1.790
P	0.309	0.422	0.116	0.139	0.100	0.544	0.257	0.619	0.096	0.080
ICC	0.896	0.765	0.919	0.940	0.831	0.921	0.942	0.944	0.821	0.798
**L5**										
Radiographic image	46.44±2.64	32.43±2.03	13.41±1.39	13.12±1.31	26.27±1.46	26.36±1.44	32.13±2.22	17.73±1.96	16.91±1.60	17.24±1.97
Printed model	46.37±2.65	32.53±1.87	13.32±1.40	13.25±1.34	26.17±1.26	26.29±1.53	32.04±2.09	17.67±1.73	17.05±1.43	17.18±1.91
T	0.716	-1.145	0.811	-0.954	0.894	0.867	0.476	0.745	-1.693	0.806
P	0.478	0.258	0.422	0.346	0.376	0.391	0.636	0.46	0.098	0.425
ICC	0.976	0.951	0.865	0.783	0.822	0.940	0.815	0.944	0.942	0.968

**Note**: **WVD**: Width of vertebral body; **APDVD**: Anteroposterior diameter of vertebral body; **LHVD**: Left height of vertebral body; **RHVD**: Right height of vertebral body; **WVC**: Width of vertebral canal; **APDVC**: Anteroposterior diameter of vertebral canal; **WRP**: Width of right pedicle; **HRP**: Height of right pedicle; **WLP**: Width of left pedicle; **HLP**: Height of left pedicle.

All paired t-test values comparing the radiographic image and 3D-printed model data of all parameters were >0.05 (C1: Table 1; C2: Table 2; C3–C7: Table 3; T1–T12: Table 4; L1–L5: Table 5). Therefore, Intraclass Correlation Coefficient (ICC) analysis was used to assess the correlation between the data from radiographic images and 3D-printed models.

Furthermore, 88.6% of all parameters of the cervical spine (Tables 1, 2 and 3), 90% of all parameters of the thoracic spine (Table 4), and 94%ofall parameters of the lumbar spine (Table 5) were >0.800. The other ICC values were <0.800 and >0.600, and none were <0.600.

## Discussion

Surgeons are constantly exploring novel internal or external fixation techniques to improve healthcare for patients. When they identify “new ideas”, surgeons should ideally test their feasibility using cadaveric models. Unfortunately, the rate of body donation for use in research is very low [4]. Furthermore, in most developing countries, particularly those with strong religious beliefs or without higher education, the rate of donation is lower than in developed countries [19, 20].

In China, universities and medical schools have been faced with an ongoing shortage of cadavers for education and research because of aspects of the Chinese culture [21];the donation rate has also been found to be low in Greece [20]. As a result, for most surgeons, it is difficult to obtain sufficient cadaveric specimens on which to test their “new ideas”.

In addition, most cadaveric specimens are stored in formalin, a storage technique that will change the shape of the bone if the specimens are stored for long periods. This is unacceptable for experiments that require accurate data.

To allow surgeons to test the feasibility of newly developed fixation techniques, we must provide accurate spine bone models. With the development of 3D digital reconstruction, it is now possible to test new fixation techniques on 3D digital images. Puchwein et al [17]studied the morphometry of the odontoid peg and its impact on ventral screws (one screw or two screws) using3D digital images; similar methods could resolve the problem caused by a lack of available cadavers. Unfortunately, digital images sometimes do not provide sufficiently accurate data. We previously imitated trans-pedicle, trans-disc oblique lumbar interbody fixation using 3D digital images. For L1/2, L2/3 and L3/4 screws, the data from 3D digital images and cadavers were similar, but the data were different for L4/5 and L5/S1 screws because the screw angles were blocked by iliac bone and by part of the L5 inferior articular process [22].

3D printing techniques can use 3D digital images to print 3Dmodels, creating the possibility of printing a morphologically accurate 3Dmodel. In our study, 214 parameters from C1 to L5were measured. The method for measuring data using radiographic images was a little different from that using the 3D-printed model because the largest width values of the vertebral body and pedicle were not in the same image; therefore, systematic error could not be avoided. To minimize this error as much as possible, we chose the best matched images for measurements, and forty-five spines were included to minimize individual error. The results showed no significant difference between the data from radiographic images and from 3D-printed models. Our results showed that 88.6% of the ICC values for the parameters of the cervical spine, 90% of the ICC values for the parameters of the thoracic spine, and 94%of the ICC values for the parameters of the lumbar spine were >0.800. These results prove the strong resemblance between data from radiographic images and 3D-printed models. The mean age of the patients from which the 45 CT scans were takenwas42.5±7.7 years (range from 31 to 54 years old), and all of the patients were adults with normal spinal structure. To decrease the error as much as possible, patients with spinal diseases were excluded.

The printed material (PLA) is not very expensive, costing approximately$150 per kilogram ($0.15 per gram). One atlas or axis model is approximately 5–10 grams, whereas other cervical through lumbar vertebrae models range from10 to 35 grams. This cost will be reduced as the method becomes more widely used.

### Limitations of this study

The 3D machine used in this study cannot print a model larger than 15 cm*15 cm*25 cm. However, if the 3D model is printed at a scale lower than 1:1, the systematic error would be increased; therefore, we chose to print each vertebra separately. Because of this, we could not measure the some parameters between two segments, such as foramen height, in this study.

Although we can provide an accurate printed spinal model using our protocol, this is simply a bony model, without any soft tissues, nerves or vessels. If the screw perforates the cortical bone and extends outside the bone into soft tissue, it will be defined as failure; therefore, the current 3D-printed model is still not suitable for some techniques that are needed to study the relationship between a screw and soft tissue. However, most spinal fixation techniques, including pedicle screw fixation, odontoid screw fixation, atlantoaxial transarticular screw fixation, lateral screw fixation, and pedicle rib screw fixation, all of which are known to be safe if the screw does not perforate more than 2 mm outside the cortex, could be studied using3D-printed models.

In the future, if we are able to use different materials to print discs, facet joints and ligaments, it may become possible to conduct biomechanical studies directly on 3D-printed models.

## Conclusion

In this study, we provide a protocol for printing accurate 3D spinal models that can be used by surgeons and researchers. This 3D-printed model is inexpensive and can easily be obtained for spinal fixation research.

## References

[1] Kabins MB, Weinstein JN. The history of vertebral screw and pedicle screw fixation. The Iowa orthopaedic journal. 1991;11:127.

[2] Yahiro MA. Comprehensive literature review. Pedicle screw fixation devices. Spine (Phila Pa 1976). 1994;19(20 Suppl):2274S–8S. Epub 1994/10/15. . 7817242 10.1097/00007632-199410151-00004

[3] Yahiro MA. Comprehensive Literature Review: Pedicle Screw Fixation Devices. Spine. 1994;19(20):2274S–8S. 7817242 10.1097/00007632-199410151-00004

[4] Boulware LE, Ratner LE, Cooper LA, LaVeist TA, Powe NR. Whole body donation for medical science: a population-based study. Clin Anat. 2004;17(7):570–7. Epub 2004/09/18. 10.1002/ca.10225 . 15376295

[5] Alvin MD, Abdullah KG, Steinmetz MP, Lubelski D, Nowacki AS, Benzel EC, et al Translaminar screw fixation in the subaxial cervical spine: quantitative laminar analysis and feasibility of unilateral and bilateral translaminar virtual screw placement. Spine (Phila Pa 1976). 2012;37(12):E745–51. Epub 2012/02/11. 10.1097/BRS.0b013e31824c70ef . 22322372

[6] Kanna PR, Shetty AP, Rajasekaran S. Anatomical feasibility of pediatric cervical pedicle screw insertion by computed tomographic morphometric evaluation of 376 pediatric cervical pedicles. Spine. 2011;36(16):1297–304. 10.1097/BRS.0b013e3181fb3c17 21289586

[7] Xia DD, Lin SL, Chen W, Shen ZH, Li Y, Wang XY, et al Computed tomography morphometric analysis of C2 translaminar screw fixation of Wright's technique and a modified technique in the pediatric cervical spine. Eur Spine J. 2014;23(3):606–12. Epub 2013/12/18. 10.1007/s00586-013-3130-9 24337233 PMC3940813

[8] Lin SL, Xia DD, Chen W, Li Y, Shen ZH, Wang XY, et al Computed tomographic morphometric analysis of the pediatric occipital condyle for occipital condyle screw placement. Spine (Phila Pa 1976). 2014;39(3):E147–52. Epub 2013/11/01. 10.1097/BRS.0000000000000105 . 24173015

[9] Robertson PA, Stewart NR. The radiologic anatomy of the lumbar and lumbosacral pedicles. Spine (Phila Pa 1976). 2000;25(6):709–15. Epub 2000/04/07. . 10752103 10.1097/00007632-200003150-00010

[10] Ji W, Wang XY, Xu HZ, Yang XD, Chi YL, Yang JS, et al The anatomic study of clival screw fixation for the craniovertebral region. Eur Spine J. 2012;21(8):1483–91. Epub 2012/02/03. 10.1007/s00586-012-2151-0 22298235 PMC3535259

[11] Vaccaro AR, Rizzolo SJ, Allardyce TJ, Ramsey M, Salvo J, Balderston RA, et al Placement of pedicle screws in the thoracic spine. Part I: Morphometric analysis of the thoracic vertebrae. J Bone Joint Surg Am. 1995;77(8):1193–9. Epub 1995/08/01. . 7642664 10.2106/00004623-199508000-00008

[12] Horn TJ, Harrysson OL. Overview of current additive manufacturing technologies and selected applications. Sci Prog. 2012;95(Pt 3):255–82. Epub 2012/10/26. . 23094325 10.3184/003685012X13420984463047 PMC10365362

[13] Haverman TM, Karagozoglu KH, Prins HJ, Schulten EA, Forouzanfar T. [Rapid prototyping: a very promising method]. Ned Tijdschr Tandheelkd. 2013;120(3):136–41. Epub 2013/04/23. . 23600178 10.5177/ntvt.2013.03.12213

[14] Webb PA. A review of rapid prototyping (RP) techniques in the medical and biomedical sector. J Med Eng Technol. 2000;24(4):149–53. Epub 2000/12/06. . 11105287 10.1080/03091900050163427

[15] Giordano RA, Wu BM, Borland SW, Cima LG, Sachs EM, Cima MJ. Mechanical properties of dense polylactic acid structures fabricated by three dimensional printing. J Biomater Sci Polym Ed. 1996;8(1):63–75. Epub 1996/01/01. . 8933291 10.1163/156856297x00588

[16] Guo SZ, Heuzey MC, Therriault D. Properties of polylactide inks for solvent-cast printing of three-dimensional freeform microstructures. Langmuir. 2014;30(4):1142–50. Epub 2014/01/15. 10.1021/la4036425 . 24410099

[17] Puchwein P, Jester B, Freytag B, Tanzer K, Maizen C, Gumpert R, et al The three-dimensional morphometry of the odontoid peg and its impact on ventral screw osteosynthesis. Bone Joint J. 2013;95-B(4):536–42. Epub 2013/03/30. doi: 10.1302/0301-620X.95B4.30949 95-B/4/536 [pii]. . 23539707 10.1302/0301-620X.95B4.30949

[18] Tian NF, Xu HZ, Wang XY, Chen QJ, Zheng LC. Morphometric comparisons between the pedicle and the pedicle rib unit in the immature Chinese thoracic spine: a computed tomographic assessment. Spine (Phila Pa 1976). 2010;35(16):1514–9. Epub 2010/05/22. 10.1097/BRS.0b013e3181c6d9ae . 20489678

[19] Asad AL, Anteby M, Garip F. Who donates their bodies to science? The combined role of gender and migration status among California whole-body donors. Soc Sci Med. 2014;106:53–8. Epub 2014/02/19. doi: 10.1016/j.socscimed.2014.01.041 S0277-9536(14)00068-9 [pii]. . 24534732 10.1016/j.socscimed.2014.01.041

[20] Halou H, Chalkias A, Mystrioti D, Iacovidou N, Vasileiou PV, Xanthos T. Evaluation of the willingness for cadaveric donation in Greece: a population-based study. Anat Sci Educ. 2013;6(1):48–55. Epub 2012/08/02. 10.1002/ase.1304 . 22851304

[21] Zhang L, Wang Y, Xiao M, Han Q, Ding J. An ethical solution to the challenges in teaching anatomy with dissection in the Chinese culture. Anat Sci Educ. 2008;1(2):56–9. Epub 2009/01/30. 10.1002/ase.15 . 19177382

[22] Wu AM, Tian NF, Wu LJ, He W, Ni WF, Wang XY, et al A radiological and cadaveric study of oblique lumbar interbody fixation in patients with normal spinal anatomy. Bone Joint J. 2013;95-B(7):977–82. Epub 2013/07/03. doi: 10.1302/0301-620X.95B7.31393 95-B/7/977 [pii]. . 23814253 10.1302/0301-620X.95B7.31393

